# Visualizing the coordination of apurinic/apyrimidinic endonuclease (APE1) and DNA polymerase β during base excision repair

**DOI:** 10.1016/j.jbc.2023.104636

**Published:** 2023-03-22

**Authors:** Max S. Fairlamb, Maria Spies, M. Todd Washington, Bret D. Freudenthal

**Affiliations:** 1 Department of Biochemistry and Molecular Biology, University of Kansas Medical Center, Kansas City, Kansas, USA; 2 Department of Biochemistry and Molecular Biology, University of Iowa Carver College of Medicine, Iowa City, Iowa, USA; 3 Department of Cancer Biology, University of Kansas Medical Center, Kansas City, Kansas, USA; 4 University of Kansas Cancer Center, Kansas City, Kansas, USA

**Keywords:** base excision repair, DNA damage, single-molecule biophysics, substrate specificity, DNA polymerase

## Abstract

Base excision repair (BER) is carried out by a series of proteins that function in a step-by-step process to identify, remove, and replace DNA damage. During BER, the DNA transitions through various intermediate states as it is processed by each DNA repair enzyme. Left unrepaired, these BER intermediates can transition into double-stranded DNA breaks and promote genome instability. Previous studies have proposed a short-lived complex consisting of the BER intermediate, the incoming enzyme, and the outgoing enzyme at each step of the BER pathway to protect the BER intermediate. The transfer of BER intermediates between enzymes, known as BER coordination or substrate channeling, remains poorly understood. Here, we utilize single-molecule total internal reflection fluorescence microscopy to investigate the mechanism of BER coordination between apurinic/apyrimidinic endonuclease 1 (APE1) and DNA polymerase β (Pol β). When preformed complexes of APE1 and the incised abasic site product (APE1 product and Pol β substrate) were subsequently bound by Pol β, the Pol β enzyme dissociated shortly after binding in most of the observations. In the events where Pol β binding was followed by APE1 dissociation during substrate channeling, Pol β remained bound for a longer period of time to allow disassociation of APE1. Our results indicate that transfer of the BER intermediate from APE1 to Pol β during BER is dependent on the dissociation kinetics of APE1 and the duration of the ternary complex on the incised abasic site.

Reactive oxygen species generate DNA damage at the alarming rate of 10^4^ lesions/cell/day (1, 2). These potentially mutagenic DNA lesions in turn promote numerous human diseases (3, 4, 5, 6). The cell’s primary defense against oxidative DNA damage is the multiprotein base excision repair (BER) pathway, Figure 1 (5, 7, 8, 9, 10, 11). BER is initiated when a damaged nucleobase is identified and bound by one of multiple types of DNA glycosylases. These DNA glycosylases bind and cleave the N-glycosidic bond of the damaged base, leaving a baseless sugar moiety known as an abasic (AP) site. This AP site is a substrate for human apurinic/apyrimidinic endonuclease 1 (APE1), which incises the DNA backbone on the 5′ side of the AP site. The resulting product, herein referred to as 5′ nick, consists of a single nucleotide gap and a one-nucleotide long 5′-deoxyribose phosphate (dRP) flap. The 5′-dRP flap is subsequently excised by the lyase activity of DNA polymerase beta (Pol β), resulting in a one-nucleotide (1-nt) gap intermediate. Pol β fills this 1-nt gap by inserting a nondamaged deoxynucleoside monophosphate resulting in a single-stranded nick on the 3′ side of the newly inserted nucleotide, referred to herein as a 3′ nick consisting of a 3′-OH and 5′-phosphate. Finally, the last enzymatic step is performed by the X-ray cross-complementing group 1 and DNA ligase III alpha heterodimer (XRCC1:Lig3α), which seals the single-stranded nick and completes the BER pathway (12, 13, 14, 15, 16, 17, 18, 19, 20, 21).

**Figure 1:**
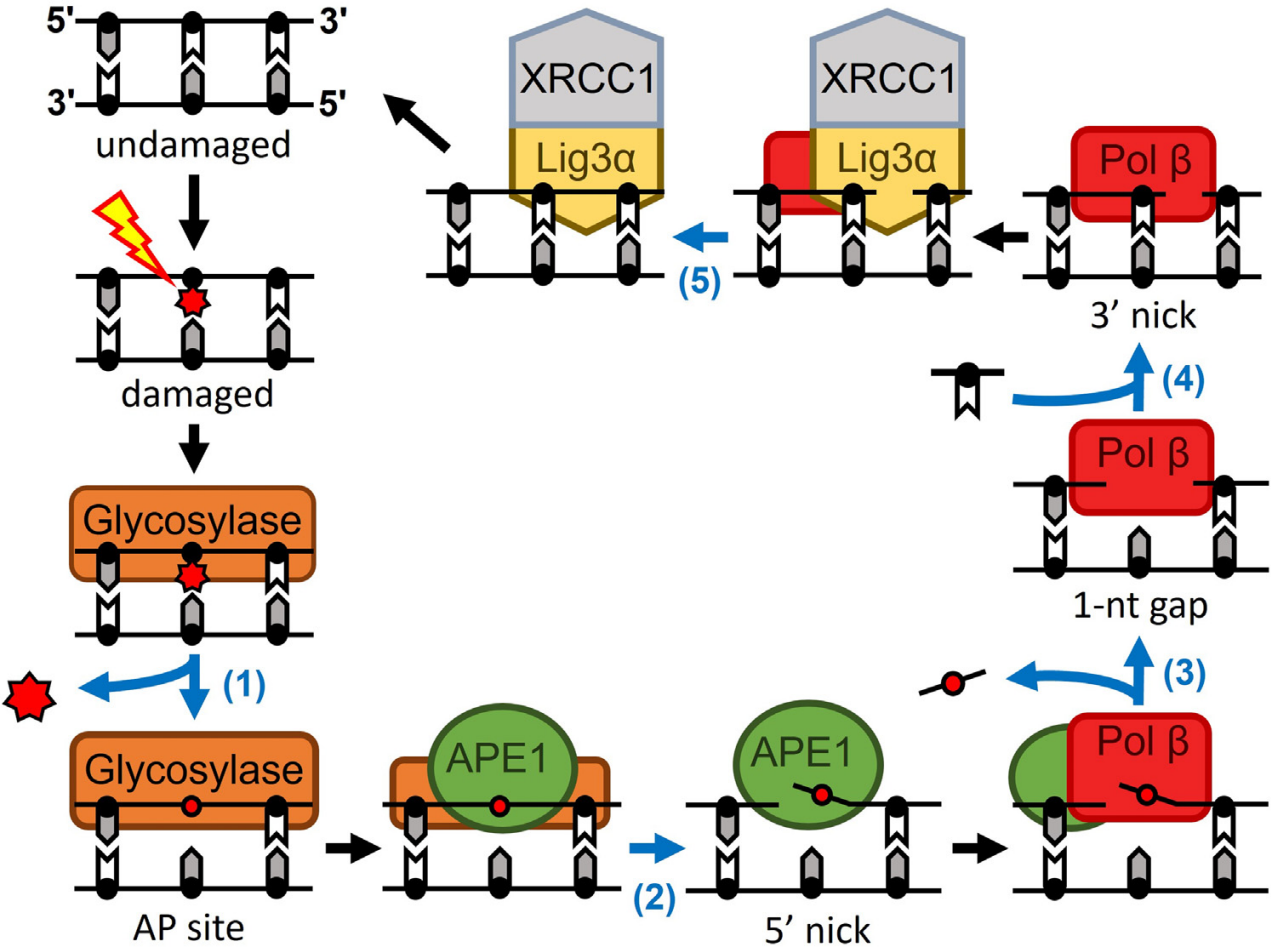
**Base excision repair pathway with each catalytic step shown with a *blue arrow*.** After the formation of DNA damage (*lightning bolt* and *red asterisk*), a DNA glycosylase cleaves the N-glycosidic bond of the damaged base (1) generating an AP site (*red circle*). APE1 cleaves the 5′ side of the AP site (2), resulting in a 5′ nick substrate containing a dRP flap. Pol β excises the dRP flap (3), resulting in a 1-nt gap substrate, and inserts a nucleotide across from the templating base (4), resulting in the 3′ nick substrate. XRCC1:Lig3α heterodimer, which ligates the single-stranded nick (5). Proposed ternary complexes are depicted between the catalytic steps of the pathway (before 2, before 3, and before 5).

The various intermediate states that the DNA damage transitions through during BER (AP site, 5′ nick, 1-nt gap, 3′ nick) are referred to as BER intermediates, Figure 1. If BER intermediates are left unrepaired, they can degrade into double-stranded DNA breaks, inhibit the progression of replicative DNA polymerases, and/or trigger programmed cell death (22, 23, 24). To stabilize the BER intermediates during repair, it has been proposed that BER enzymes remain bound to their respective BER intermediate product until the next enzyme in the pathway binds (Fig. 1) (25, 26, 27, 28, 29).This transfer of BER intermediates between BER enzymes is referred to as BER coordination or substrate channeling and has been described as analogous to runners passing a DNA baton (30). The importance of this mechanism is highlighted by previous studies that have shown that disruption of BER coordination can result in diminished BER efficiency and genomic instability (31, 32).

Consistent with the model of substrate channeling during BER coordination, previous kinetic and protein foot printing studies indicate that transfer of the 5′ nick DNA substrate from APE1 to Pol β involves the formation of a three-member complex composed of APE1, Pol β, and the 5′ nick BER intermediate that is APE1’s product and Pol β′s substrate (28, 33, 34). We refer to this transient complex formation as the APE1–Pol β–5′ nick ternary complex. The details of how APE1–Pol β–5′ nick ternary complex formation promotes transference of the 5′ nick BER intermediate between APE1 and Pol β remains unclear. Part of the challenge in characterizing this BER coordination mechanism has been the difficulty of resolving the timing of association and dissociation steps that occur during the formation of APE1–Pol β–5′ nick ternary complexes. For example, work from Dr Samuel Wilson’s lab demonstrated that an AP site could be incised by APE1, transferred to Pol β, and further processed into downstream BER intermediates within a 10-s time frame (28). However, these experiments were unable to resolve how long the APE1–Pol β–5′ nick ternary complex remained intact or the order of APE1 and Pol β binding and release from the BER intermediate. These details could provide critical insight into the mechanism of BER coordination between APE1 and Pol β.

To investigate substrate channeling during BER coordination, we utilized single-molecule total internal reflection fluorescence (TIRF) microscopy to visualize the sequence and timing of association and disassociation steps that occur during the formation of APE1–Pol β–5′ nick ternary complexes. Our results indicate that APE1 and Pol β both form the most stable interactions with their respective substrates and products relative to other BER intermediates in the pathway. We also find that the association of Pol β to a preformed APE1–5′ nick complex was most frequently followed by rapid Pol β dissociation. In the majority of events where Pol β association was followed by APE1 dissociation, the APE1–Pol β–5′ nick ternary complex was longer-lived. Based on these findings, we show that, in the absence of other BER factors, DNA hand-off events involving APE1 and Pol β reflect the probability that APE1 product release will occur before Pol β dissociates from the complex.

## Results

### APE1 and Pol β binary interactions with various BER intermediates

**Figure 2:**
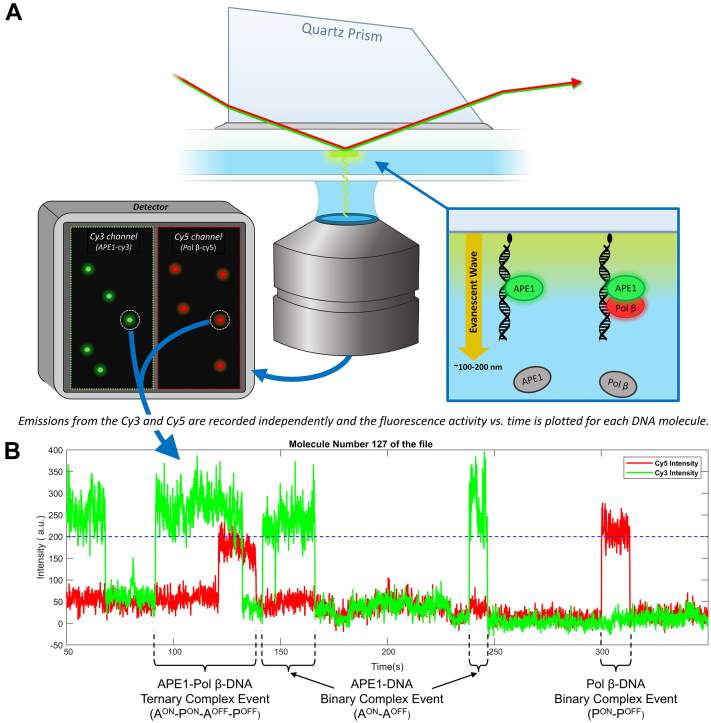
**Single-moleculetotal internalreflection fluorescence experimental setup.***A*, The 532- and 640-nm excitation lasers (*red and green arrows*) are directed to the imaged region of the sample chamber *via* a quartz prism. Total internal reflection of the excitation beams produces an evanescent wave (*yellow arrow*) that excites fluorophores located within ∼100 to 200 nm of the slide surface. Visible fluorescent spots indicate binding interactions between a single immobilized DNA molecule and an excited APE1-Cy3 (*green oval*) and/or Pol β-Cy5 (*red oval*) molecules. *B*, fluorescence trajectories that depict the association of APE1-Cy3 or Pol β-Cy5 with an immobilized DNA molecule are indicated by an increase in fluorescence intensity in the Cy3 or Cy5 channel, respectively, while dissociation is indicated by a decrease in intensity back to baseline. Binary complex events involve association and dissociation of a single protein from the DNA substrate. Ternary complex events involve the association of both APE1-Cy3 and Pol β-Cy5 before the dissociation of protein.

To understand how the state of the BER intermediate affects APE1 binding specificity, we first measured the time that APE1 remained bound to each BER intermediate using single-molecule TIRF microscopy (Fig. 2*A*) (35). We refer to binding events that consist of one protein and one DNA molecule as binary events. In each experiment, fluorescently labeled protein was flowed into a sample chamber containing immobilized DNA that mimicked a specific BER intermediate (AP site, 5′ nick, 1-nt gap, 3′ nick, or undamaged DNA) and binary events were recorded. Fluorescent trajectories, which depict the fluorescence intensity observed at a single immobilized DNA molecule as a function of time (Fig. 2*B*), were then extracted from the recordings. The time that each binary complex remained intact (*i.e.*, the complex lifetime) was used to construct a cumulative residence time distribution (CRTD) plot, which represents the percent of observed protein–DNA complexes that remained bound as a function of time (36).

**Figure 3:**
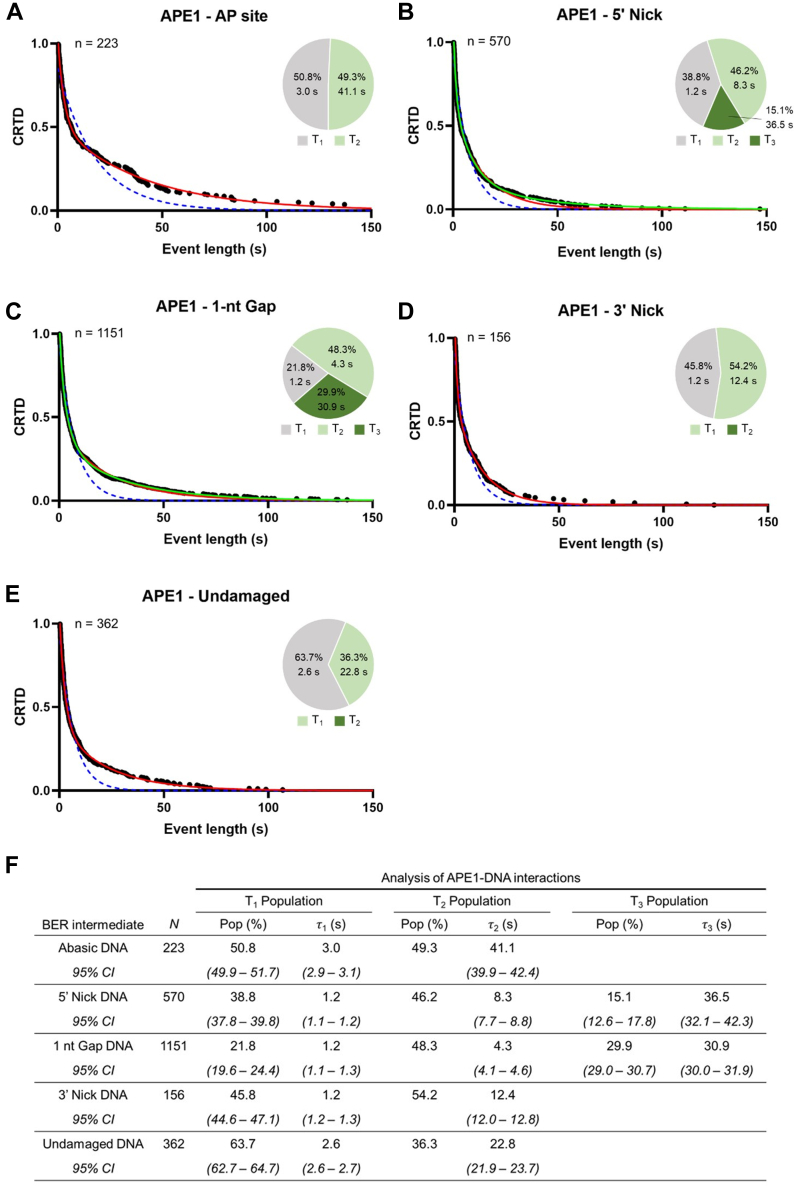
**Cumulative residence time distribution (CRTD)*****versus*****time plot of binary complex lifetimes measured from APE1 binding events.** The DNA substrates containing an (*A*) AP site, (*B*) 5′ nick, (*C*) 1-nt gap, (*D*) 3′ nick, and (*E*) undamaged DNA are shown with experimental data represented by *black circles*, and single, double, and triple exponential fits are depicted as *dotted blue*, *solid red*, and *solid green lines*, respectively. Pie charts show the relative fraction of molecules associated with the T_1_, T_2_, and T_3_ populations. *F*, estimates for the mean lifetime and dissociation rate constants for APE1 on BER intermediates. N represents the total number of observed counts. Pop is the coefficient of the exponential terms obtained from the fits and represents the relative fraction of all observed objects represented by the population. *τ*_i_ represents the mean lifetime of the respective population. The *lower* and *upper* bounds of the 95% confidence intervals (CI) are shown for each reported parameter.

The CRTD plots describing the dissociation kinetics of binary complexes between APE1 and each of the BER intermediates are presented in Figure 3. Each CRTD plot was fit to an exponential decay model to yield the mean lifetime of the complex. For each interaction tested, the features of the CRTD plots were best described by either a double or triple exponential model (Fig. S1*A*). This indicates the existence of multiple independent populations of APE1–DNA binding events occurring within each dataset. For example, the APE1–AP site CRTD plot was best represented by a double exponential decay model, indicating the existence of a short- and long-lived population of APE1–AP site binding events that we denote as T_1,[APE1]_ and T_2,[APE1]_ (Fig. 3*A*). In contrast, the APE1–5′ nick CRTD plot was best represented by a triple exponential decay model (Fig. S1*A*). This indicates the existence of three populations of APE1–5′ nick binding events, which we denote as T_1,[APE1]_, T_2,[APE1]_, and T_3,[APE1]_, with each population having a different mean lifetime. The average lifetime of the short-lived T_1_ population is similar for each BER intermediate tested, suggesting that this population of binding events reflects an unstable protein–DNA interaction that is not necessarily related to the specific features of the DNA damage intermediate. However, the timescale of the longer-lived T_2_ and T_3_ populations is dependent on the BER intermediate tested, which suggests that these populations relate to specific interactions between the protein and the DNA lesion (Fig. 3). Therefore, to analyze differences in the interactions between APE1 and each BER intermediate, we focus our analysis on the mean lifetimes of the longer-lived T_2_ and T_3_ event populations.

The T_2_ population of APE1–AP site binding events lasted an average of 41.1 s (Fig. 3*A*). Since catalysis is inhibited with EDTA, this long lifetime likely reflects APE1 interactions with a substrate that it cannot catalytically convert to product. Meanwhile, interactions between APE1 and the incised product, 5′ nick, exhibited a T_2_ population with an average lifetime of 8.3 s and a T_3_ population of 36.5 s (Fig. 3*B*). APE1 binding event populations with downstream BER intermediates were generally shorter-lived. Binding events between APE1 and the 1-nt gap BER intermediate exhibited T_2_ and T_3_ populations with average lifetimes of 4.3 s and 30.9 s, respectively (Fig. 3*C*). APE1 interactions with the 3′ nick intermediate and undamaged DNA had T2 populations with mean lifetimes of 12.4 s and 22.8 s, respectively (Fig. 3, *D* and *E*). Altogether, these findings suggest that the binding specificity of APE1 to the DNA lesion may promote APE1 association at the proper stage of the BER pathway.

**Figure 4:**
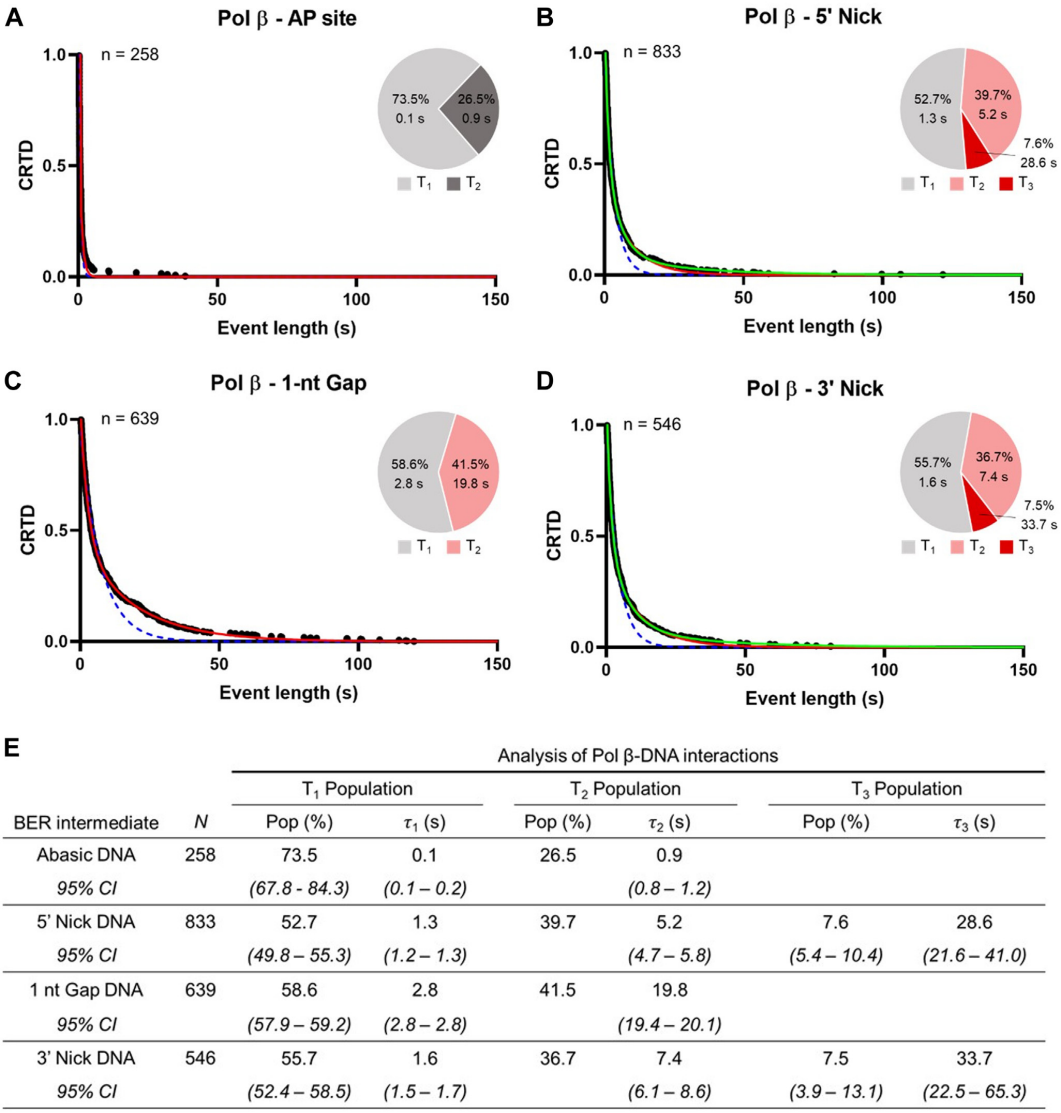
**Cumulative residence time distribution (CRTD)*****versus*****time plot of binary complex lifetimes measured from Pol β binding events.** The DNA substrates containing an (*A*) AP site, (*B*) 5′ nick, (*C*) 1-nt gap, and (*D*) 3′ nick are shown with experimental data represented by *black circles*, and single, double, and triple exponential fits are depicted as *dotted blue*, *solid red*, and *solid green lines*, respectively. Pie charts show the relative fraction of molecules associated with the T_1_, T_2_, and T_3_ populations. *E*, estimates for the mean lifetime and dissociation rate constants for Pol β on BER intermediates. N represents the total number of observed counts. Pop is the coefficient of the exponential terms obtained from the fits and represents the relative fraction of all observed objects represented by the population. τ_i_ represents the mean lifetime of the respective population. The *lower* and *upper* bounds of the 95% confidence intervals (CI) are shown for each reported parameter.

Similar to the APE1–DNA interactions, binary Pol β–DNA binding events for each BER intermediate were also characterized (Figs. 4 and S1*B*). Initial attempts to record binary interactions between Pol β and DNA containing an abasic site or no DNA damage (undamaged DNA) resulted in very few stable complexes that lasted longer than ∼1 s (Fig. 4*A*). Consistent with previous findings, this suggests that these intermediates are not preferred substrates of Pol β due to the lack of a free 5′ phosphate (34). In contrast, interactions between Pol β and DNA containing 5′ nick, 1-nt gap, and 3′ nick were readily observed. As with APE1–DNA interactions, these datasets were best described by either two or three kinetic processes (T_1,[Polβ]_, T_2,[Polβ]_, and if applicable T_3,[Polβ]_) with mean lifetimes that differed by an order of magnitude (Fig. 4). Similar to APE1 the T_1_ binding events are not specific to the BER intermediate, and therefore we are focusing on the T_2_ and T_3_ binding events. Binding interactions between Pol β and the 5′ nick (which is the first substrate of Pol β in BER) had T_2_ and T_3_ populations with mean lifetimes of 5.2 s and 28.6 s, respectively (Fig. 4*B*). Meanwhile, Pol β interactions with the 1-nt gap (which is the product formed after Pol β lyase activity and the substrate for Pol β gap filling) exhibited a T_2_ population with a mean lifetime of 19.8 s (Fig. 4*C*). Finally, binding events between Pol β and the 3′ nick (which is the final product of Pol β) exhibited T_2_ and T_3_ populations with mean lifetimes of 7.4 s and 33.7 s, respectively (Fig. 4*D*). The Pol β–AP site binary events indicated that Pol β–AP site complexes are relatively short-lived with a mean T_2_ lifetime of approximately 0.9 s (Fig. 4). Altogether, these data indicate that Pol β–DNA binding is highly selective for BER intermediates that contain single-stranded nicks (5′ nick, 1-nt gap, and 3′ nick). The short-lived interactions between Pol β and undamaged DNA or DNA containing an AP site suggests a lack of lesion engagement by Pol β, resulting in only fast dissociating events being observed. Similar to our findings with APE1, these results suggest that the specific DNA binding properties of Pol β may help ensure Pol β associates with the DNA at the correct stage of BER and does not interfere with upstream and downstream steps of the pathway.

### Assembly of APE1–Pol β–5′ nick ternary complexes and BER coordination

**Figure 5:**
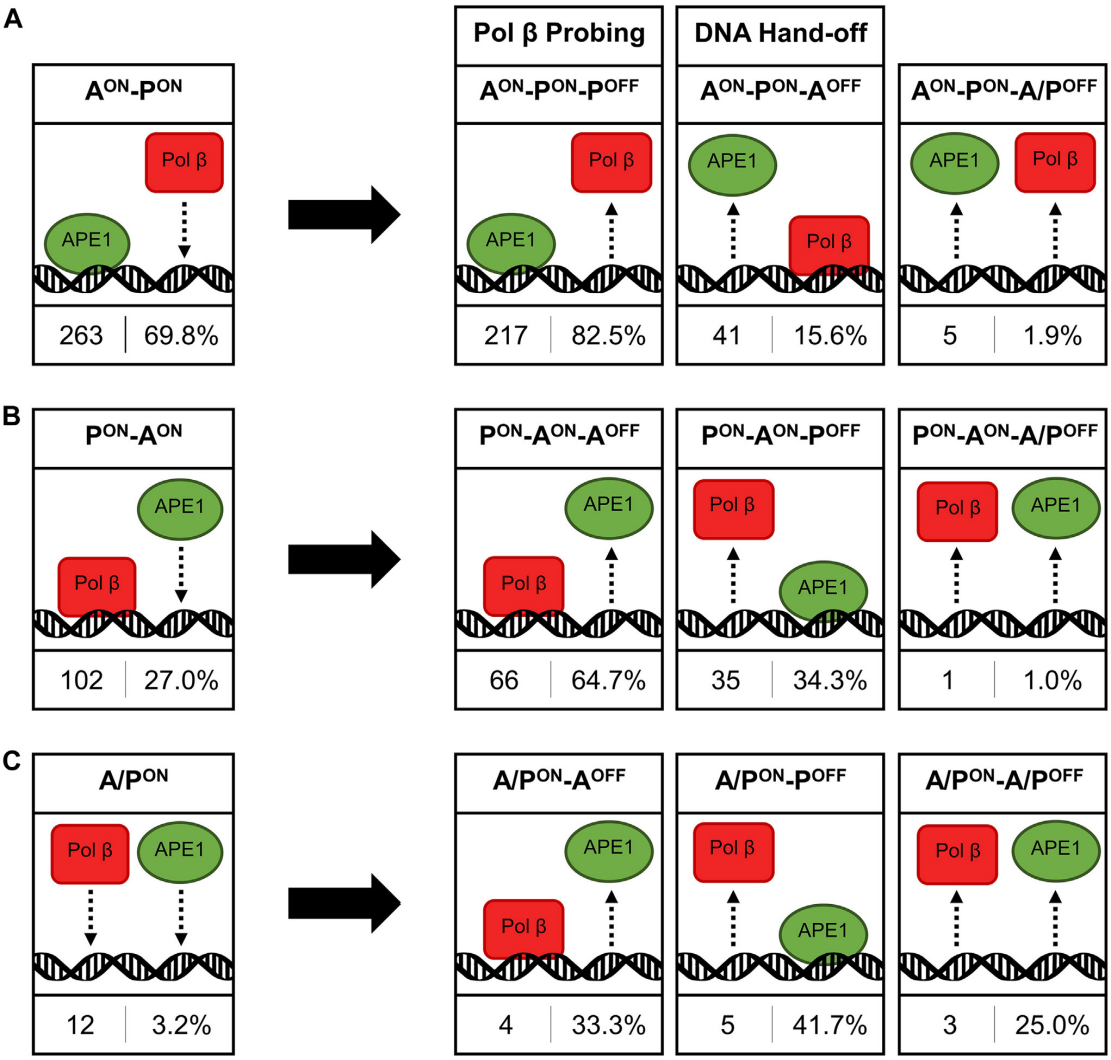
**Ternary Complex Event Tree.***A*, of the 377 ternary complex formations recorded, 263 ternary complexes formed when a preformed APE1–5′ nick binary complex was bound by Pol β (A^ON^-P^ON^), (*B*)102 formed when a preformed Pol β–5′ nick binary complex was bound by APE1 (P^ON^-A^ON^), and (*C*) 12 formed when both proteins associated within the same 0.1-s frame (A/P^ON^). The relative occurrence of each possible disassembly sequence (A^OFF^, P^OFF^, or A/P^OFF^) is displayed to the *right* of the *black arrows*. Pol β probing events (A^ON^-P^ON^- P^OFF^) and DNA hand-off events (A^ON^-P^ON^-A^OFF^) are specifically indicated.

According to the model of BER coordination between APE1 and Pol β, an APE1–Pol β–5′ nick ternary complex is formed before the dissociation of APE1 (30). To understand how these ternary complexes assemble, we used single-molecule TIRF microscopy to record the order of APE1-Cy3 and Pol β-Cy5 binding to immobilized 5′ nick BER intermediates. A representative fluorescence trajectory of an APE1–Pol β–5′ nick ternary complex formation is shown in Figure 2*B*. Of the 377 ternary complex formations, 263 (69.8%) formed when a preformed APE1–5′ nick binary complex was bound by Pol β (A^ON^-P^ON^) (Fig. 5*A*). These events reflect the proper directionality of the BER pathway. In addition, we observed 102 ternary complexes (27.0%) that formed upon APE1 association to a preformed Pol β–5′ nick binary complex (P^ON^-A^ON^, Fig. 5*B*) and 12 ternary complexes (3.2%) that formed upon both APE1 and Pol β associating within the same frame (A/P^ON^, Fig. 5*C*). The low frequency of observed A/P^ON^ events relative to A^ON^-P^ON^ and P^ON^-A^ON^ events suggests that APE1–Pol β–5′ nick DNA ternary complexes primarily form through sequential binding of APE1 and Pol β. Thus, these results provide further evidence that APE1–Pol β–5′ nick ternary complexes primarily assemble through the independent association of each enzyme to the DNA substrate as opposed to the association of preformed APE1–Pol β complex.

**Figure 6:**
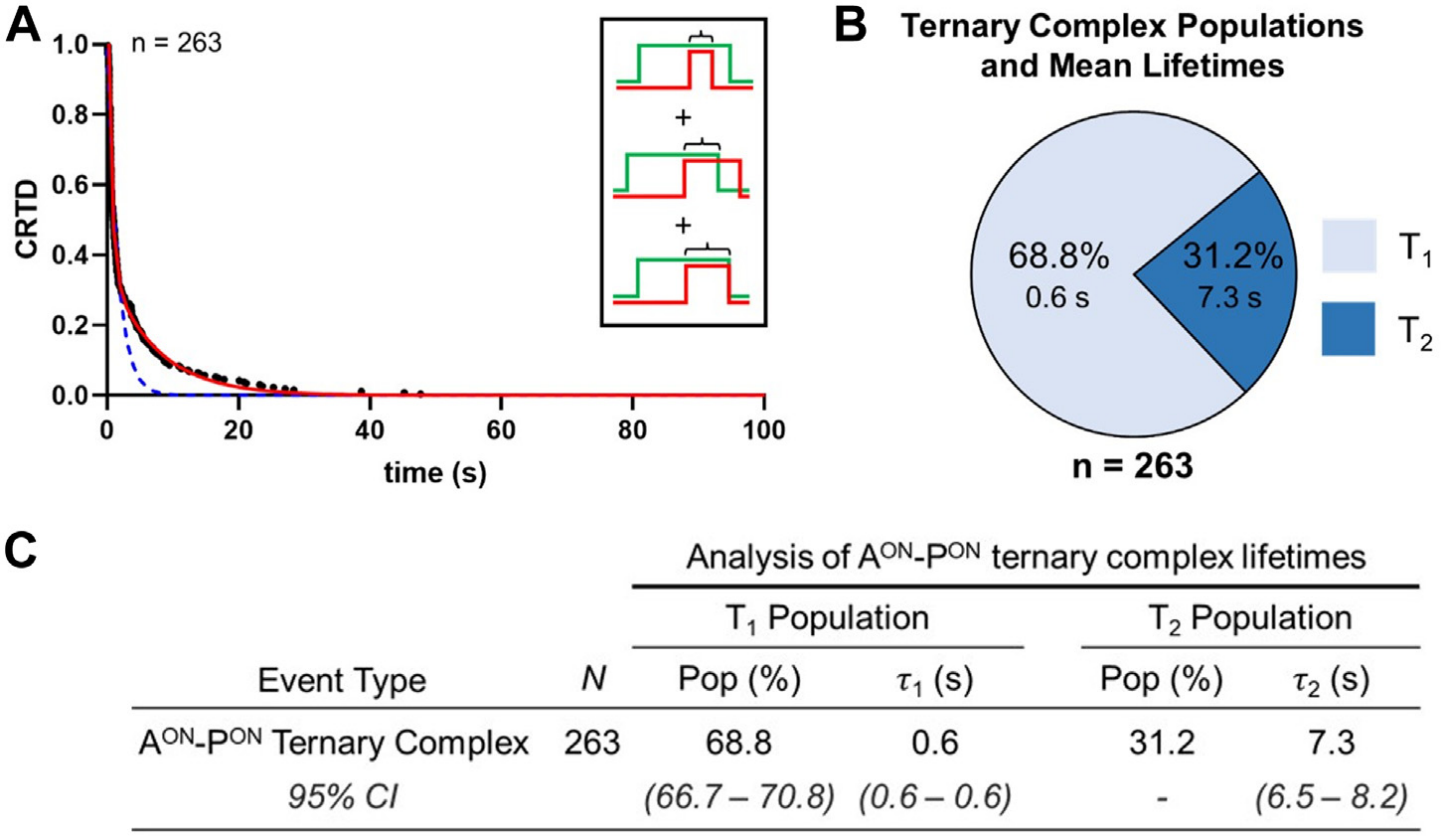
**Ternary lifetime of APE1–Pol β–5′ nick ternary complexes.***A*, log–log plot of cumulative residence time distribution (CRTD) *versus* time for the lifetime of APE1–Pol β–5′ nick ternary complexes that formed when Pol β associated with a preformed APE1–5′ nick binary complex (A^ON^-P^ON^, n = 263). *Black circles* represent experimental data, and single and double exponential fits are shown in *dashed blue* and *solid red lines*, respectively. The inset shows the different types of ternary complexes traces we observed in these data with Pol β in red and APE1 in *green*. *B*, the fraction of ternary complexes that participate in the fast dissociating (T_1_) and slow dissociating (T_2_) processes are represented with *light blue* and *dark blue* segments of a pie chart, respectively. N represents the total number of observed counts. *C*, N represents the total number of observed counts. Pop is the coefficient of the exponential terms obtained from the fits and represents the relative fraction of all observed objects represented by the population. τ_i_ represents the mean lifetime of the respective population. The *lower* and *upper* bounds of the 95% confidence intervals (CI) are shown for each reported parameter.

The canonical BER coordination model proposes that, after incision of the AP-site by APE1, the 5′ nick product is transferred to Pol β through the formation of an APE1–Pol β–5′ nick ternary complex. To determine the stability of the APE1–Pol β–5′ nick ternary complex, we measured the duration that each ternary complex remained intact (*i.e.*, the ternary complex lifetime) for ternary events in which Pol β associated to a preformed APE1–5′ nick complex (A^ON^-P^ON^) (Fig. 6, inset). The average lifetime of these 263 ternary complexes was then quantified using CRTD analysis (Fig. 6*A*). Fitting of the CRTD plot was best described by two kinetic processes (T_1_ and T_2_), suggesting the existence of a short-lived and long-lived subpopulation of ternary complexes. The relative fraction of ternary complexes that dissociated according to the T_1_ and T_2_ processes is shown in Figure 6, *B* and *C*. In the majority of ternary events (68.8%), the mean lifetime of the ternary complex was relatively short-lived (T_1_ = 0.6 s), whereas the remaining 31.2% of ternary events exhibited a longer-lived ternary complex (T_2_ = 7.3 s) (Fig. 6). This finding led us to investigate if T_1_ and T_2_ populations reflect different sequences of ternary complex disassembly.

In order to distinguish whether the T_1_ and T_2_ populations in Figure 6 reflect different sequences of APE1 and Pol β dissociation, we first categorized ternary events based on the order of enzyme disassociation. Of the 263 A^ON^-P^ON^ ternary events, 217 events (82.5%) ended with a Pol β disassociation step (A^ON^-P^ON^-P^OFF^, Fig. 5*A*). These events, which we refer to as Pol β probing events, reflect unsuccessful transfer of the DNA substrate. In many cases, after the formation of the APE1–5′ nick DNA complex, multiple repeated Pol β probing events were observed before the eventual dissociation of APE1. In contrast, 41 events (15.6%) ended with an APE1 disassociation step (A^ON^-P^ON^-A^OFF^, Fig. 5*A*). These events, which we refer to as DNA hand-off events, are true to the directionality of the BER pathway and reflect the transfer of the 5′ nick APE1 product from APE1 to Pol β. Only five ternary complexes (1.9%) ended with both APE1 and Pol β dissociating within the same 0.1-s frame window (A^ON^-P^ON^-A/P^OFF^, Fig. 5*A*). These rare events likely represent the simultaneous dissociation of APE1 and Pol β from the immobilized 5′ nick DNA. These results provide evidence that APE1–Pol β–5′ nick ternary complexes primarily disassemble through the independent dissociation of each enzyme. Furthermore, these results suggest that the transfer of APE1’s product from APE1 to Pol β (DNA hand-off) can indeed occur under the conditions tested. Importantly, this is consistent with ensemble studies that showed APE1 to Pol β hand-off occurring on a similar time scale (28). However, association of Pol β to the APE1–5′ nick complex most frequently results in Pol β dissociation and unsuccessful transfer of the DNA (Pol β probing).

In addition to the 263 ternary complexes that formed upon Pol β association to an APE1–DNA complex (A^ON^-P^ON^, Fig. 5*A*), we observed 102 ternary complexes (27.0%) that formed upon APE1 association to a Pol β–5′ nick complex (P^ON^-A^ON^, Fig. 5*B*). Of these, 35 events (34.3%) ended with a Pol β disassociation step (P^ON^-A^ON^-P^OFF^). These events are counteractive to the directionality of the BER pathway, reflecting the transfer of the 5′ nick APE1 product from Pol β to APE1. Another 66 events (64.7%) ended with an APE1 disassociation step (P^ON^-A^ON^-A^OFF^), which correspond to the association and subsequent dissociation of APE1 from Pol β–5′ nick complexes. The remaining one event (1.0%) ended with both proteins dissociating within the same frame (P^ON^-A^ON^-A/P^OFF^, Fig. 5*C*). These results provide further evidence that APE1–Pol β–5′ nick ternary complexes primarily assemble and disassemble through sequential association and dissociation of each enzyme. Furthermore, the finding that A^ON^-P^ON^ events primarily end with Pol β dissociation (A^ON^-P^ON^-P^OFF^), and P^ON^-A^ON^ events primarily end with APE1 dissociation (P^ON^-A^ON^-A^OFF^), indicates that of the order of disassembly is dependent on the order in which the proteins assemble on the DNA substrate. This suggests that the initially bound protein forms more stable DNA interactions than the incoming protein. This may reflect the site-specific DNA binding of the initially bound protein to the 5′ nick, which may obstruct accessibility for the incoming protein.

**Figure 7:**
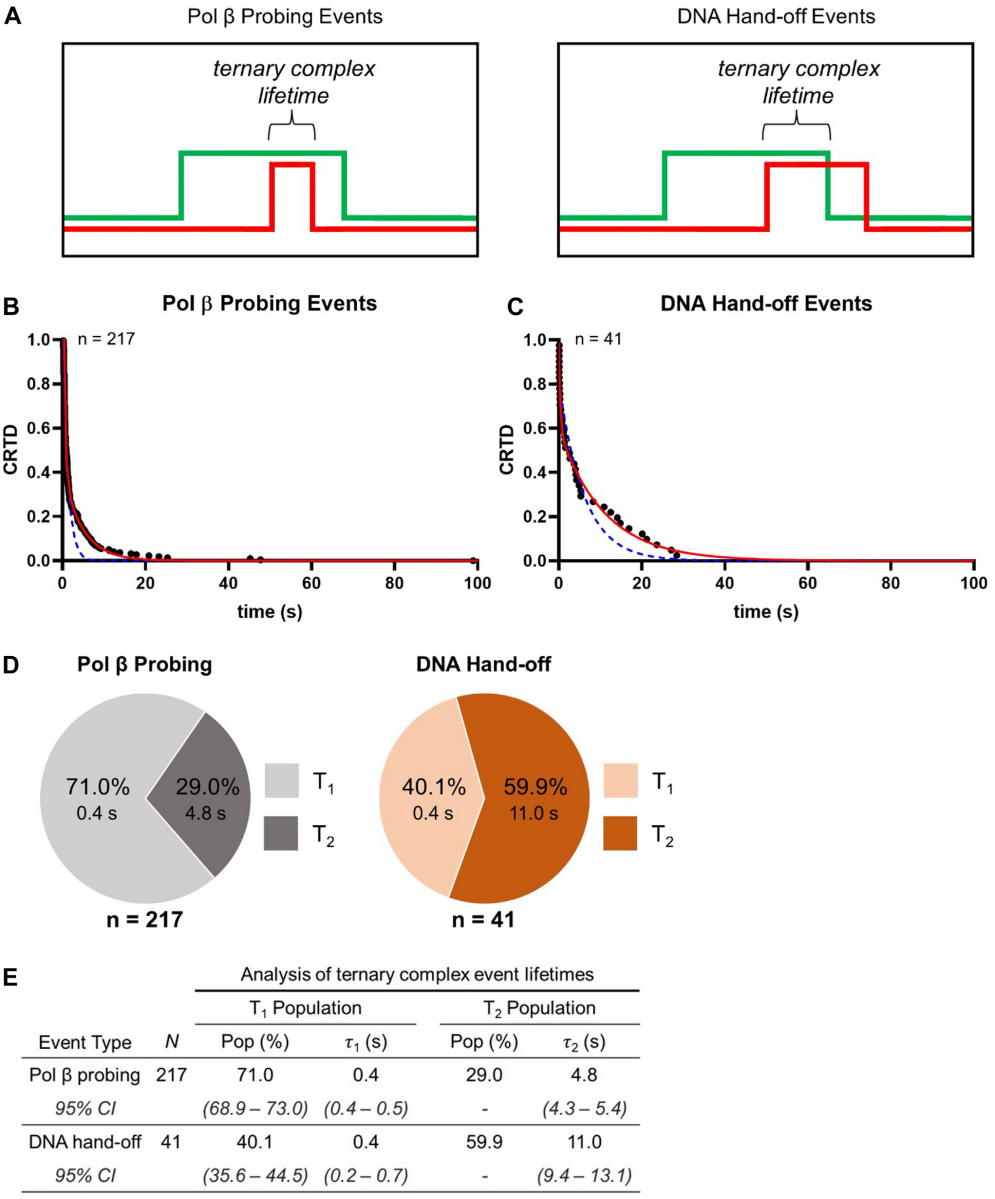
**DNA hand-off** ***versus*** **Pol β probing during ternary complex formation.***A*, the lifetime of a ternary complex is the duration that both proteins are simultaneously bound to the immobilized DNA. The order of complex disassembly defines the Pol β probing (*left panel*) *versus* DNA hand-off (*right panel*). Pol β is shown as a *red line* and APE1 a *green line*. *B* and *C*, the log-log plot of cumulative residence time distribution (CRTD) *versus* time plot of ternary complex lifetimes measured from Pol β probing events and DNA hand-off events. Experimental data are represented by *black circles*, and single and double exponential fits are depicted as *dashed blue* and *solid red lines*, respectively. *D*, the fraction of ternary complexes that participate in the fast dissociating (T_1_) and slow dissociating (T_2_) Poisson processes observed for Pol β probing events (*gray*) and DNA hand-off events (*orange*) is represented with pie charts. N represents the total number of observed counts. *E*, pop is the coefficient of the exponential terms obtained from the fits and represents the relative fraction of all observed objects represented by the population. τ_i_ represents the mean lifetime of the respective population. The *lower* and *upper* bounds of the 95% confidence intervals (CI) are shown for each reported parameter.

To better understand what may allow an APE1–Pol β–5′ nick ternary complex to result in DNA hand-off *versus* Pol β probing, we measured the ternary complex lifetime of each Pol β probing event and DNA hand-off (Fig. 7*A*). The average lifetimes of ternary complexes for Pol β probing events (A^ON^-P^ON^-P^OFF^) and DNA hand-off events (A^ON^-P^ON^-A^OFF^) were then quantified using CRTD analysis (Fig. 7, *B* and *C*). Both the Pol β probing and DNA hand-off CRTD plots were best described by double exponential models reflecting two kinetic processes, suggesting the existence of a short-lived (T_1_) and long-lived (T_2_) population of ternary complexes. In the majority of Pol β probing events (71.0%), the mean lifetime of the ternary complex was relatively short-lived (0.4 s), while the majority of DNA hand-off events (59.9%) exhibited a longer-lived ternary complex (11.0 s) (Fig. 7, *D* and *E*). These results indicate that, after association of Pol β to the APE1–5′ nick complex, the ternary complex is more likely to end with DNA hand-off (APE1 dissociation) if Pol β remains bound to the APE1–5′ nick complex for longer than 0.4 s, specifically around 11 s. This suggests that the order of ternary complex disassembly (Pol β probing *versus* DNA hand-off) is dependent on the disassociation of APE1 and the duration of the ternary complex on the 5′ nick.

## Discussion

In order to progress through the steps of BER, the correct BER enzyme should be bound to the BER intermediate at the relevant stage in the pathway. To analyze differences in the interactions between the BER proteins and each BER intermediate, we utilized single-molecule TIRF to measure the lifetime of individual protein–DNA binding events. Each protein–DNA interaction was best represented by at least two populations of events, with each population exhibiting a different average lifetime. For binary APE1–DNA interactions and Pol β–DNA interactions, the shortest-lived population (T_1_) was similar for each BER intermediate tested, suggesting this population of binding events reflects an unstable protein–DNA interaction unrelated to the specific structure of the DNA damage. Although the current approach does not allow us to discern the cause for the multiple binding event populations, the mean lifetimes of the longer-lived T_2_ and T_3_ event populations were dependent on the BER intermediate tested, which suggests that these populations relate to specific interactions between the protein and the DNA lesion.

Both APE1 and Pol β formed the most stable complexes with the BER intermediates that mimicked the canonical substrates of the respective enzyme. Binary interactions between APE1 and the AP site substrate and 5′ nick product exhibited the longest-lived binding event populations compared with downstream BER intermediates (Fig. 3). Similarly, Pol β exhibited binding event populations with the longest mean lifetime for 5′ nick, 1-nt gap, and 3′ nick, which reflect the canonical substrates and products of Pol β (Fig. 4). Interestingly, compared with APE1, Pol β forms much weaker complexes with BER intermediates that are not canonical substrates or products (AP site and undamaged). This finding may reflect the difference in general affinity that APE1 and Pol β have for undamaged duplex DNA, as APE1 has been shown to interact with nonspecific DNA with more stability than Pol β (34, 37). Altogether, our results demonstrate a high binding specificity between the BER enzymes and their respective substrates. This is consistent with previous work that suggested that the ordered association of each BER enzyme could be explained by the intrinsic substrate binding properties of each enzyme (34). Furthermore, lower affinity between BER enzymes and upstream or downstream BER intermediates in the pathway could minimize the competitive inhibition caused by the association of BER enzymes that are not relevant to the current stage of BER. Thus, the binding specificity of each BER enzyme for specific BER intermediates likely contributes to the proper directionality of BER, helping to ensure the correct proteins are bound at the right times for efficient progression of the pathway.

After association of Pol β to the APE1–5′ nick complex, the Pol β active site must gain access to the 5′ nick lesion, which is precluded by the active site of APE1. This transfer could occur spontaneously after APE1 independently dissociates from the 5′ nick, or it could involve a coordinated eviction of APE1 from the 5′ nick lesion by Pol β and/or other BER factors. Here we identified two main types of ternary events: Pol β probing events consisting of Pol β association and disassociation from an APE1–5′ nick complex, and DNA hand-off events consisting of Pol β association with an APE1–5′ nick complex followed by APE1 dissociation (Figure 5, Figure 6, Figure 7). Surprisingly, we observed over 5-fold more Pol β probing events than DNA hand-off events. These findings suggest that Pol β alone is not capable of efficiently evicting APE1 from the 5′ nick lesion. Furthermore, in the majority of Pol β probing events, the ternary complex was relatively short-lived (1.1 s), while in the majority of DNA hand-off events the ternary complex was longer-lived (11 s). This suggests that the likelihood a given ternary event will end with DNA hand-off is largely dependent on the dissociation of APE1 and the duration that Pol β remains bound near the APE1–5′ nick complex. This finding, along with the high frequency of observed Pol β probing events relative to DNA hand-off events, leads us to hypothesize that the hand-off events observed here between APE1 and Pol β reflect the probability that APE1 product release will occur before Pol β dissociates from the complex. It is possible that frequent probing of the APE1–5′ nick complex by Pol β could be a means to capture the lesion immediately after APE1 dissociation. Pol β has been shown to search a region of approximately 24 base pairs through a DNA hopping mechanism (37). Therefore, in the context of the cell where there are longer spans of DNA flanking the APE1–5′ nick complex, Pol β could remain in the vicinity of the lesion for longer, extending the amount of time Pol β can wait for APE1 to dissociate and allow for frequent probing of the 5′ nick lesion. Overall, these results provide the first direct visualization and analysis of BER coordination events and provide novel mechanistic insight into the efficiency and timing of APE1–Pol β coordination.

While we have focused here on the ability of APE1 and Pol β to transfer the 5′ nick BER intermediate, it is likely that the coordination of efficient BER is facilitated by additional contributing factors as observed in multiple cellular studies characterizing the temporal dynamics of BER factors (38, 39). For example, the scaffolding protein X-ray repair cross-complementing 1 (XRCC1) has been previously shown to be capable of simultaneously binding multiple proteins involved with BER including APE1 (32), Pol β (17), and Lig3α (14). It is likely that scaffolding proteins such as XRCC1 might bridge the two proteins, keeping Pol β near the lesion long enough for APE1 to dissociate. Moreover, XRCC1 interactions with APE1 have been shown to promote APE1 product release (32). Thus, XRCC1 may act to promote APE1 release when Pol β is ready to accept the BER intermediate. The single-molecule TIRF approach developed here could be extended to examine the effects of XRCC1 and other BER factors on the formation of ternary complexes with BER intermediates. Finally, it may be possible that APE1–Pol β coordination is facilitated by catalytic cofactors, which were omitted from the experiment. For example, completion of the gap-filling step requires binding of a free nucleotide in the Pol β polymerase active site, which is followed by a conformational change in the catalytic core (22). Molecular dynamic simulations suggest that this conformational change could reduce the predicted APE1–Pol β interface by ∼10%, causing a decrease in APE1–Pol β affinity upon association of a free nucleotide. It is plausible that this could trigger the timely dissociation of APE1. Similar to previous studies, the components required for enzymatic activity were sequestered in these studies to inhibit turnover of the substrate during experimentation (33, 34, 40). Future studies will explore whether the inclusion of free nucleotide or catalytic metals affects the lifetime of ternary complexes or the order of APE1 and Pol β disassembly.

## Experimental procedures

### DNA substrates

Oligonucleotides were obtained from IDT. Annealing reactions were performed by mixing the oligonucleotides in a 1:1.2 ratio of biotinylated template to unbiotinylated complement(s). Reaction mixtures were heated to 95 °C for 5 min and then allowed to cool slowly to 4 °C. To generate the 25-mer duplex DNA used to capture APE1–DNA and Pol β–DNA binary complex formations, a 5′-biotin end-labeled 25-mer template (5′-[biotin]TGT GTG GAA TAC AGT GAG CGC AAC G-3′) was annealed with the respective oligo(s) described below to yield duplex substrates that mimicked each of the canonical BER intermediates. The AP site BER intermediate was prepared from the template and a complement sequence that contained a centrally placed tetrahydrofuran (THF) group (5′-CGT TGC GCT CA[THF] TGT ATT CCA CAC A-3′). The 5′ nick BER intermediate was prepared from the template, a downstream 11-mer primer (5′-CGT TGC GCT CA-3′), and an upstream 13-mer primer containing a dRP group and terminal phosphate (phos) (5′-[phos][dRP]TGT ATT CCA CAC A-3′). The 1-nt gap BER intermediate was generated by the same approach using an upstream 13-mer primer that lacked the 5′-dRP (5′-[phos]TGT ATT CCA CAC A-3′). The 3′ nick BER intermediate was generated using the same upstream 13-mer primer and a downstream 12-mer primer (5′-CGT TGC GCT CAC-3′). A 3′-Cy5-labeled version of the 25-mer template was used to collect binary events between Pol β and AP site DNA. The 40-mer immobilized DNA substrate used for capturing APE1–Pol β–5′ nick ternary complex formations was prepared from a 5′-biotinylated and 3′-Cy5 labeled template (5′-[biotin]ATG CAT GTT GTG TGG AAT ACA GTG AGC GCA ACG CAA TCA C[Cy5]-3′), a 21-mer downstream primer (5′-[phos][dRP]T GTA TTC CAC ACA ACA TGC AT-3′), and an 18-mer upstream primer (5′-GTG ATT GCG TTG CGC TCA-3′).

### Protein expression, purification, and fluorescent labeling

Human wildtype APE1 and wildtype Pol β were expressed and purified as described (41, 42). Briefly, protein was overexpressed in BL21(DE3)plysS *Escherichia coli* cells (Invitrogen) bearing a pET28a plasmid (Genscript) that encoded either APE1 or Pol β. Cells were grown in 2xYT at 37 °C, expression was induced with IPTG, and harvested cells were lysed *via* sonication. Protein was purified from the cell lysate *via* heparin, cation exchange, and gel filtration resins using an FPLC (ATKA-Pure). Purity and concentration of the resulting protein solution was confirmed by SDS-PAGE analysis and by absorbance at 280 nm.

APE1 and Pol β were fluorescently labeled with Cy3 and Cy5 NHS ester dye, respectively. APE1 was dialyzed in labeling buffer containing 50 mM Hepes, pH 7.05, and 150 mM sodium chloride and concentrated to 15 mg/ml. While avoiding exposure to light, an Amersham Cy3 monoreactive NHS ester dye pack (GE healthcare) was resuspended with labeling buffer and mixed with enough purified APE1 to yield a 100 μl solution of 10 mg/ml APE1. The reaction was rotated slowly in darkness for 16 h at 4 °C. Excess dye was removed by running the labeling reaction over a cation exchange column using an FPLC (ATKA-Pure). Fluorescent labeling of Pol β followed the same procedure using an Amersham Cy5 monoreactive NHS ester dye pack (GE healthcare). Labeling efficiency was determined by calculating the molar ratio of dye to protein within the solution by measuring absorbance at 280 nm and 530 nm for APE1-Cy3 or 649 nm for Pol β-Cy5. For both Cy3-labeled APE1 and Cy5-labeled Pol β, labeling efficiencies were between approximately 2.1 to 2.4 labels per protein. Single-use aliquots of fluorescently labeled proteins were flash-frozen with liquid nitrogen and stored until use at −80 °C for up to 1 month.

### Single-molecule imaging and data collection

The single-molecule data were collected on a prism-type TIRF microscope, which has been described (35). Briefly, excitation lasers (OBIS Coherent) with wavelengths of 532 and 640 nm are merged using a series of dichroic mirrors (Shamrock) and directed to the stage of an inverted microscope (Olympus America, Inc) using broadband mirrors (Thorlabs). The 532- and 640-nm excitation lasers were operated at 60 mW and 30 mW power, respectively. The excitation beams pass through a Pellin-Broca prism (Eksma Optics), which modulates the trajectory of the beams and generates an evanescent field of excitation within the visualized region of the sample chamber. Cy3 and Cy5 emissions were observed using a 60× objective lens (Olympus) and split using an Optosplit-III emission image spitter (Cairn). Recordings were captured using an IXON ULTRA 897 EMCCD camera (Andor) at 10 frames per second.

TIRF experiments were performed using quartz sample chambers coated with Biotin-PEG-SVA (Laysan Bio), which were cleaned and prepared as described (43, 44). The sample chamber was first flushed with 0.2 mg/ml neutravidin (ThermoFisher) in buffer (50 mM Hepes, pH7.4, 150 nM NaCl) and allowed to sit for 3 min, then unbound neutravidin was flushed out with buffer. All subsequent steps used imaging buffer containing 50 mM Hepes, pH 7.4, 150 mM sodium chloride, 0.1 mg/ml bovine serum albumin, 0.8% glucose, and 2.5 mM EDTA. For fluorophore longevity and stability, imaging buffer also contained approximately 10 mM Trolox (Cayman), 0.04 mg/ml Catalase (Sigma-Aldrich), and 1 mg/ml Glucose Oxidase (Sigma-Aldrich) as described (45). Unless otherwise stated, binary protein–DNA events were collected by first incubating a 350 pM solution of 25-mer DNA in the sample chamber for 3 min, flushing the unbound DNA, and then flowing 0.1 nM of each fluorescently labeled protein into the chamber. Binding events between proteins and immobilized DNA were recorded for 20 min at room temperature, and fluorescent trajectories were extracted from the recordings. Experiments designed to capture the formation of APE1–Pol β–5′ nick ternary complexes were performed similarly, except a 15 pM solution of Cy5-labeled 40-mer DNA was incubated in the sample chamber. Also, after starting the recording, the Cy5-labeled DNA was bleached by flushing the chamber with buffer lacking the oxygen scavenging system before adding 1.5 nM of each fluorescently labeled protein.

Fluorescence trajectories, which display the measured intensity of each fluorophore at each immobilized DNA substrate as a function of time, were generated from recordings as described (45). Sustained increases in fluorescence intensity that reflected APE1-Cy3 or Pol β-Cy5 binding events to immobilized DNA and satisfied the selection rules as defined in (46) were selected for analysis and idealized with QuB (47) (University of Buffalo). For experiments that utilized Cy5-labeled DNA, only the fluorescence trajectories that exhibited Cy5 signal from the beginning of the recording were analyzed. Events were then organized into an event catalog using the Kinetic Event Resolving Algorithm (KERA) software package, which yields the relative frequency of each type of event observed and the duration between each association and disassociation step within each event (48).

### CRTD analysis

To quantify the dissociation kinetics of a particular complex formation, the lifetimes of all observed complexes (*i.e.*, the interval of time between the association and dissociation steps) were compiled into a CRTD plot (36). The CRTD plot can be interpreted as a type of normalized survival curve that describes the fraction of objects remaining as a function of time when the start of all events is synchronized to time = 0. This can be modeled as a Poisson process, which is defined as a population of discrete events where the exact time between individual events (such as interval of time between protein–DNA complex association and disassociation) is random, but the average time between events is constant, as reviewed in (49). The probability of observing a protein–DNA complex with a lifetime (*T*) that is greater than or equal to a given time (*t*) decreases exponentially as time *t* is increased. This can be described by Equation 1:

(1) $$P\left(T\geq t\right)=\sum_{i=1}^{N_{p}}f_{i}e^{-t/\tau_{i}}$$

where *P(T ≥ t)* is the probability that the duration of a randomly selected event will be at least as long as time *t.* The term *N*_*p*_ denotes the number of distinct Poisson processes that contribute to the dissociation kinetics of the overall population, and *f*_*i*_ represents the relative fraction of all observed objects represented by each subpopulation (*i*). The term *τ*_i_ is the average lifetime of the complex, which can be used to derive the dissociation rate constant k_d,i_ (*τ*_i_ = k_d,i_^−1^). To determine the minimum number of Poisson processes that adequately captures all of the features of a given dataset, CRTD plots were fit to single (*N*_*p*_ = 1), double (*N*_*p*_ = 2), and triple (*N*_*p*_ = 3) exponential models and the most likely *N*_*p*_ value was determined using the extra sum-of-squares F test performed using GraphPad Prism version 8.2.1 for Windows (GraphPad Software) (50). From this analysis, we found that each dataset analyzed was best described by either a double or triple exponential decay model.

## Data availability

All data are available in the main text or the supporting information and supporting tables.

## Supporting information

This article contains supporting information.

## Conflict of interest

The authors declare that they have no conflicts of interest with the contents of this article.
