# Supplementary material for: Visualizing the coordination of apurinic/apyrimidinic endonuclease (APE1) and DNA polymerase β during base excision repair
Source: J Biol Chem. 2023 Mar 22;299(5):104636. doi: 10.1016/j.jbc.2023.104636 (PMC10148159; doi:10.1016/j.jbc.2023.104636)
Supplement: Supplemental Figure S1 [file mmc1.pdf]

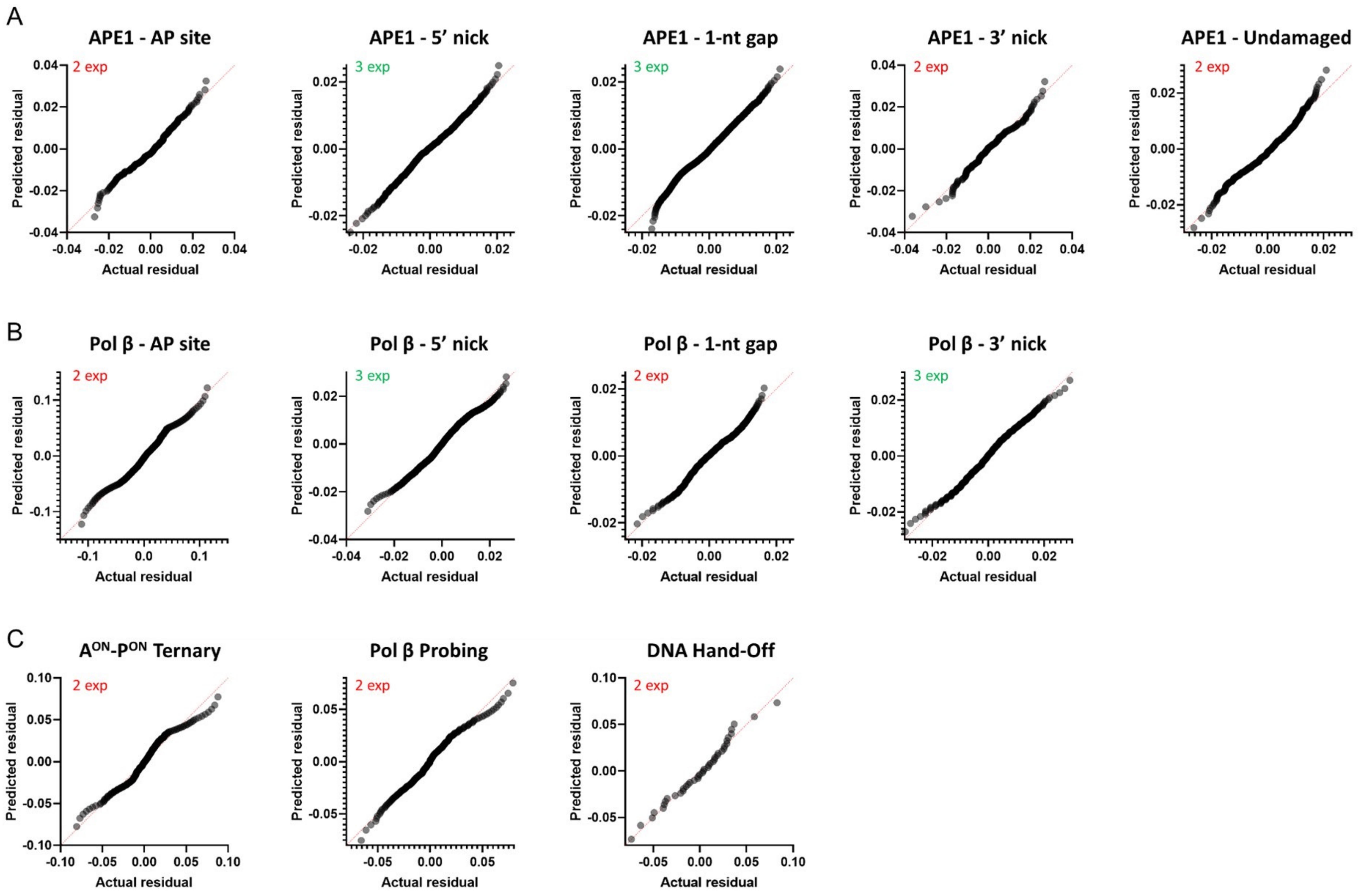

Supplemental Figure 1A. Residual QQ plots of CRTD plot fits for datasets representing (A) APE1 binary interactions shown in Figure 3, (B) Pol β binary interactions shown in Figure 4, and (C) APE1-Pol β-5' nick DNA ternary interactions shown in Figure 7.
